# Dynamic evolution and mechanism of myocardial glucose metabolism in different functional phenotypes of diabetic cardiomyopathy — a study based on ^18^ F-FDG microPET myocardial metabolic imaging

**DOI:** 10.1186/s13098-023-01038-5

**Published:** 2023-04-01

**Authors:** Xiaoliang Shao, Yaqi Liu, Mingge Zhou, Min Xu, Yuqi Chen, Hongbo Huang, Jianguo Lin, Yuetao Wang

**Affiliations:** 1 Department of Nuclear Medicine, the Third Affiliated Hospital of Soochow University, Changzhou, 213003 China; 2 Clinical Translational Institute for Nuclear Medicine and Molecular Imaging, Soochow University, Changzhou, 213003 China; 3 Echocardiography Division in Department of Cardiology, the Third Affiliated Hospital of Soochow University, Changzhou, 213003 China; 4 NHC Key Laboratory of Nuclear Medicine, Jiangsu Key Laboratory of Molecular Nuclear Medicine, Jiangsu Institute of Nuclear Medicine, Wuxi, 214063 China

**Keywords:** Diabetic cardiomyopathy, Myocardial glucose metabolism, Cardiac function, microPET, Mechanism

## Abstract

**Purpose:**

To use ^18^ F-FDG microPET dynamic imaging to preliminarily identify the changes of myocardial glucose metabolism corresponding to different functional phenotypes of diabetic cardiomyopathy (DCM) in mice and elucidate their relationships.

**Methods:**

Left ventricular function was measured by echocardiography in C57BL/KsJ-db/db (db/db) mice and their controls at 8, 12, 16, and 20 weeks of age to divide DCM stages and functional phenotypes. Myocardial histopathology was used to verify the staging accuracy and list-mode microPET dynamic imaging was conducted. The myocardial metabolic rate of glucose (MRglu) and the glucose uptake rate constant (Ki) were derived via Patlak graphical analysis, and the differences in myocardial glucose metabolism levels in different DCM stages were compared. The key proteins involved in myocardial glucose metabolism signaling pathway were analyzed by Western blotting to elucidate the underlying mechanism of abnormal glucose metabolism in DCM.

**Results:**

Compared with the controls, the ratio of early diastolic transmitral flow velocity to early diastolic mitral annular tissue velocity (E/e’) of db/db mice was significantly increased from the age of 12 weeks, while the left ventricular ejection fraction (LVEF) was significantly decreased from the age of 16 weeks (all *P* < 0.05). Based on the staging criteria, 8 and 12 weeks (8/12w) db/db mice were in DCM stage 1 (diastolic dysfunction with normal LVEF), and 16 and 20 weeks (16/20w) db/db mice were in DCM stage 2/3 (diastolic and systolic dysfunction). The degree of myocardial fibrosis, glycogen deposition and ultrastructural damage in 16/20w db/db mice were more obvious than those in 8/12w group. The myocardial MRglu, Ki of db/db mice in 8/12w group or 16/20w group were significantly lower than those in the control group (all *P* < 0.05), while the myocardial standard uptake value (SUV) was not significantly reduced in the 8/12w group compared with the control group (*P* > 0.05). MRglu and SUV were moderately negatively correlated with the E/e’ ratio (r=-0.539 and − 0.512, *P* = 0.007 and 0.011), which were not significantly correlated with LVEF (*P* > 0.05). Meanwhile, Ki was not significantly correlated with LVEF or E/e’ ratio. The decreased expression of glucose transporter (GLUT) -4 in db/db mice preceded GLUT-1 and was accompanied by decreased phosphorylated AMP-activated protein kinase (p-AMPK) expression. Myocardial MRglu, Ki and SUV were significantly positively correlated with the expression of GLUT-4 (MRglu: r = 0.537; Ki: r = 0.818; SUV: r = 0.491; *P* = 0.000 ~ 0.046), but there was no significant correlation with GLUT-1 expression (*P* = 0.238 ~ 0.780).

**Conclusions:**

During the progression of DCM, with the changes of left ventricular functional phenotype, abnormal and dynamic changes of myocardial glucose metabolism can occur in the early stage.

**Supplementary Information:**

The online version contains supplementary material available at 10.1186/s13098-023-01038-5.

## Introduction

Diabetes is an important risk factor for heart failure. The incidence of heart failure in patients with diabetes is as high as 19–26% [1], which is 2.4 times and 5.1 times higher than normal men and women, respectively [2]. The risk of heart failure increases by 8% for every 1% increase in glycated hemoglobin (HbA1c) [3]. Even if blood pressure is normal and blood glucose is well controlled, nearly 50% of diabetic patients still have varying degrees of abnormal cardiac function [4]. Diabetic cardiomyopathy (DCM) is a disease characterized by ventricular dysfunction and hypertrophy, independent of hypertension, ischemia, or coronary artery disease, and is the main cause of abnormal cardiac function and increased incidence of heart failure in diabetes [4]. In 2011, Maisch et al. [5] classified DCM into 4 stages according to the dynamic evolution of left ventricular diastolic and systolic functions and defined different functional phenotypes during the progression of DCM (stage 1: diastolic dysfunction with normal ejection fraction, stage 2: diastolic and systolic dysfunction, stage 3/4: clinically overt heart disease with microvascular disease or coronary atherosclerosis). However, due to the complex mechanisms underlying the evolution from DCM to heart failure, the mechanisms underlying the changes in each functional phenotype are not fully understood, and there is still a lack of clinically recognized effective prevention and treatment methods [6].

Heart is a high energy consumption organ. 60–80% of the energy required by myocardium are generated from the β-oxidation of fatty acids, and 20–40% are from the oxidation of glucose, ketone bodies and lactic acid. The energy metabolism substrates maintain a dynamic balance through the “Randle Cycle” [7, 8]. Changes in myocardial metabolic pathways can lead to abnormal cardiac function and structure. Heart failure is a pathological state due to insufficient myocardial energy supply or imbalance of substrate metabolism [9]. In diabetes, myocardial glucose utilization is significantly decreased and fatty acid oxidation is enhanced [4, 6, 10]. This shift in substrate utilization could be a major driver of cardiac dysfunction that is a hallmark of the diabetic syndrome [11]. ^18^ F-deoxyglucose (FDG) is a clinically widely used molecular probe that reflects myocardial glucose metabolism. In the past, this technique is often used to delineate the pathophysiological process and elucidate the mechanism of DCM [12]. Brom et al. [13] studied early DCM rats and found that the insulin-mediated myocardial glucose utilization was reduced by 66%, which was associated with strong left ventricular function. However, some studies [14, 15] using microPET dynamic imaging found that the myocardial glucose metabolism rate in the early stage of DCM was significantly increased, and they believed that the metabolic disorder usually preceded the change of cardiac function. Therefore, the relationship between myocardial glucose metabolism level and cardiac function during the progression of DCM is still controversial, and the correspondence between glucose metabolism disorders and different functional phenotypes are still unclear. Therefore, in this study C57BL/KsJ-db/db mice (db/db) and their controls were used to determine cardiac functions by echocardiography and DCM functional phenotypes. The differences of myocardial glucose metabolism level, myocardial histopathology, ultrastructure under electron microscope and myocardial glucose metabolism related protein expression level in DCM with different functional phenotypes were observed, and the relationships and mechanisms were explored.

## Materials and methods

### Experimental animals

8, 12, 16, and 20-week-old C57BL/KsJ-db/db (db/db) male mice and the age-matched littermates of non-diabetic C57BL/KsJ-db/+ (db/+) mice were used in this study. All mice were purchased from GemPharmatech Ltd (Nanjing, China). The db/db mice were kept in separate cages with 2 mice/cage, and the db/+ mice were kept with 4 mice/cage, with 12/12 h light/dark cycle, free access to food and water. All experiments were performed according to the practical animal care guidelines and under protocols approved by the Experimental Animal Ethics Committee of Soochow University.

### Glucose tolerance test (GTT) and insulin tolerance test (ITT)

GTT procedure: mice were fasted for 16 h with free access to water. Glucose (1.2 g/kg) was intraperitoneally injected, and blood glucose levels were measured by tail clipping before injection, and at 15 min, 30 min, 60 min, and 120 min after injection. ITT procedure: mice were fasted for 4 h with free access to water. Insulin (0.8 U/kg) was injected intraperitoneally, and blood glucose levels were measured by tail clipping before injection and at 15 min, 30 min, 45 min, and 60 min after injection. Blood glucose was measured using a Roche ACCU-CHEK portable blood glucose meter (Performa).

### Echocardiography measurements

Echocardiography was performed using a GE VIVID E95 cardiac ultrasound detector with a 6S-D probe. The center frequency was 10 MHz, the maximum frequency was 12 MHz, and the image depth was 10 mm. Two-dimensional, M-mode, flow Doppler and tissue Doppler modes were used for image acquisition, and at least five consecutive cardiac cycles were acquired for each image. Left ventricular end-diastolic diameter (LVEDD), end-systolic diameter (LVESD) and ejection fraction (LVEF, %) can be measured from M-mode images. Doppler blood flow measurement in the apical four-chamber view was used to record the E peak of the forward flow of the mitral valve (rapid diastole), and tissue Doppler was used to record the early diastolic motion velocity of mitral annulus e’. The E/e’ ratio can be calculated.

### ^18^ F-FDG microPET myocardial dynamic imaging and image analysis

The mice fasted for 12 h before imaging with free access to water. Imaging was performed using the Inveon microPET imaging system (Siemens, Germany), and gas anesthesia was performed using the Matrx anesthesia machine and isoflurane (Midmark, United States). The mice were placed prone and fixed on the examination bed and were anesthetized with continued 1.5-2.0% isoflurane and 400 ml/min oxygen flow. ^18^ F-FDG (0.2–0.3 mCi, 50–75 ul) was injected via the tail vein, and dynamic 60-min images were acquired in list-mode. List-mode images were sorted into 43 frames (1 s × 15, 5 s × 9, 30 s × 8, 300 s × 11) and reconstructed using 3-dimensional ordered-subset expectation maximization. Images were analyzed using PMOD 4.2 (PMOD Technologies, Switzerland). The left ventricular myocardium’s volume of interest (VOI) was manually delineated slice by slice using the images from 55 min. As previously reported [16], VOI was placed over the inferior vena cava (cylinder between the renal branches and diaphragm) to generate blood-pool time-activity curves. Myocardium time-activity curves were fitted to a Patlak kinetic model using the blood-pool VOI as image-derived input function. The myocardial metabolism rate of glucose (MRglu) and glucose uptake rate constant (Ki) were derived via Patlak graphical analysis of FDG kinetics [17]. Myocardial MRglu is calculated by MRglu = Ki × Glu / LC, where Glu is the average blood glucose concentration, and LC is the lumped constant, which is assumed to be 1.0 for myocardium [18]. The myocardial SUVmean of the left ventricular was calculated by the PMOD 4.2.

### Histopathological evaluation

After imaging, the mice were sacrificed under anesthesia, and the heart was removed. The heart was cut in half by performing a transverse slice between the atrioventricular sulcus and the apex. The sample was fixed in 10% neutral formaldehyde for 24 h, embedded in paraffin, and stained with hematoxylin and eosin (HE), Masson trichrome and Periodic Acid-Schiff (PAS). HE, Masson’s trichrome and PAS staining were performed according to the manufacturer’s instructions. The semi-quantification analysis of myocardial fibrosis was determined with ImageJ version 1.6 (NIH, United States) and calculated the percentage of collagen fibers (blue) in Masson’s trichrome-stained images (×200). The semi-quantification analysis of myocardial PAS staining images was performed according to the intensity of the staining reaction (negative: -, weak: +, moderate: ++, strong: +++, intense: ++++) [19].

### Transmission electron microscopy

The myocardial tissue specimen (about 1 mm^3^) was put into 2.5% glutaraldehyde electron microscope fixative solution, fixed at 4 °C for 2 to 4 h with glutaraldehyde-osmic acid double fixation method; then, the specimen was dehydrated using gradient acetone and embedded in epoxy resin. Ultrathin 60–80 nm sections were cut and stained with 2% uranyl acetate saturated alcohol solution and lead citrate. The slides were observed and analyzed under the transmission electron microscope (HT7700, Hitachi).

### Western blotting

About 50 mg of myocardial tissue was homogenized in RIPA lysis buffer. The supernatant was collected after centrifugation. The protein concentration was measured with BCA kit (Aidlab Biotechnologies Ltd, Beijing, China). 20 µg of protein from each sample was loaded on SDS-PAGE gel, transferred to PVDF membrane by semi-dry transfer method, and stained with Ponceau red. The membrane was blocked with TBS solution containing 5% nonfat milk at room temperature for 2 h, and incubated with primary antibodies at 4 °C overnight. The primary antibodies used in this study included anti-glucose transporter-1 (GLUT-1) antibody (Abcam/ab652), anti-GLUT-4 antibody (Abcam/ab33780), anti-AMP-activated protein kinase (AMPK) antibody (Abcam/ab3760), anti-phosphorylated AMPK (p-AMPK) antibody (CST/2535) and anti-GAPDH antibody (Abcam/ab8245). After being washed with TBST buffer three times, the membrane was incubated with secondary antibody at room temperature for 2 h. After washing, the membrane was developed with chemiluminescence reagent and imaged with a fluorescence chemiluminescence gel imaging system. The IOD (integrated optical density) of each band was calculated by the Quantity One version 4.62 gel densitometry analysis software. GLUT-1 and 4 to loading control (GAPDH) IOD ratio were used as a semiquantitative measure of glucose transporter expression. The IOD ratio of p-AMPK to AMPK was used as a semiquantitative measure of phosphorylated AMPK expression.

### Statistical analysis

Statistical analysis was performed using SPSS 26.0 statistical software (IBM, USA). Kolmogorov-Smirnov first tested the measurement data to see if they obeyed normal distribution. Normally distributed measurement data were expressed as mean ± standard deviation, and non-normally distributed data were described as median (interquartile range). Count data were expressed as numbers or percentages. Two-sample independent t-test or one-way analysis of variance (one-way ANOVA) was used to compare normally distributed data, and Mann-Whitney U rank sum test was used to compare non-normally distributed data. *P* value correction was performed for the multiple tests by the Bonferroni method. Repeated measurement data were analyzed using the Analysis of Variance and sphericity test. Chi-square test was used to compare the percentage data. The relationship between two continuous variables was analyzed by Pearson correlation, and the relationship between rank data was analyzed by Spearman correlation. All *P* values were two-sided, and *P* < 0.05 was considered statistically significant.

## Results

### Dynamic changes of cardiac function in diabetic mice

As shown in Tables 1, although the LVEF of 8w and 12w db/db mice were reduced comparing with that in the control group, the difference was not statistically significant (*P* = 0.101 and 0.191); while the LVEF of 16w and 20w db/db mice were significantly reduced comparing with those in control group and 8w, 12w db/db groups (*P* = 0.000 ~ 0.024). The E/e’ of db/db mice was significantly increased comparing with that of control group from 12 weeks of age. The E/e’ of 16w and 20w db/db mice was significantly higher than that of control group and 8w, 12w db/db mice (*P* = 0.001 ~ 0.040). The findings suggest that left ventricular diastolic dysfunction precedes systolic dysfunction. Based on the definition of DCM functional phenotype [1, 5, 20], the 8w and 12w db/db mice were in DCM stage 1 (diastolic dysfunction with normal LVEF), and the 16w and 20w db/db mice were in DCM stage 2/3 (diastolic and systolic dysfunction). Thus, we combined 8w and 12w db/db mice into one group (8/12w) and 16w and 20w into another group (16/20w) to analyze their corresponding histopathology, myocardial glucose metabolism, and protein expression levels related to glucose metabolism.

**Table 1:** Dynamic changes of left ventricular function in diabetic mice

	Body weight (g)	FBG (mmol/L)	LVEF (%)	E/e’
Control (n = 8)	25.7 ± 2.3	7.0 ± 2.0	73.5 ± 3.3	17.3 ± 3.2
8w (n = 4)	45.9 ± 3.8	14.8 ± 1.5	70.0 ± 1.4	19.8 ± 2.6
^a^ *P*	< 0.001^*^	0.008^*^	0.101	0.184
12w (n = 4)	50.1 ± 4.3	25.1 ± 4.7	70.8 ± 5.4	21.2 ± 1.9
^a^ *P*	< 0.001^*^	< 0.001^*^	0.191	0.040^*^
^b^ *P*	< 0.012^#^	0.003^#^	0.752	0.482
16w (n = 4)	51.7 ± 5.1	25.9 ± 4.3	64.3 ± 2.2	24.5 ± 4.3
^a^ *P*	< 0.001^*^	< 0.001^*^	< 0.001^*^	0.001^*^
^b^ *P*	0.003^#^	0.002^#^	0.024^#^	0.036^#^
^c^ *P*	0.346	0.795	0.012^&^	0.142
20w (n = 4)	47.8 ± 4.2	25.7 ± 8.0	60.8 ± 2.8	24.5 ± 1.4
^a^ *P*	< 0.001^*^	< 0.001^*^	< 0.001^*^	0.001^*^
^b^ *P*	0.410	0.002^#^	0.001^#^	0.035^#^
^c^ *P*	0.273	0.852	< 0.001^&^	0.137
^d^ *P*	0.098	0.942	0.151	0.984

Note:

1. FBG: fasting blood glucose; LVEF: left ventricular ejection fraction; E/e’: the ratio of early diastolic transmitral flow velocity to early diastolic mitral annular tissue velocity

2. ^a^*P*: *P* value compared to Control; ^b^*P*: *P* value compared to 8w; ^c^*P*: *P* value compared to 12w; ^d^*P*: *P* value compared to 16w; the differences are significant (*P* < 0.05) when compared with control group (^*^), 8w group (^#^), and 12w group (^&^)

### Changes of myocardial histopathology and ultrastructure in different DCM stages

**Fig. 1:**
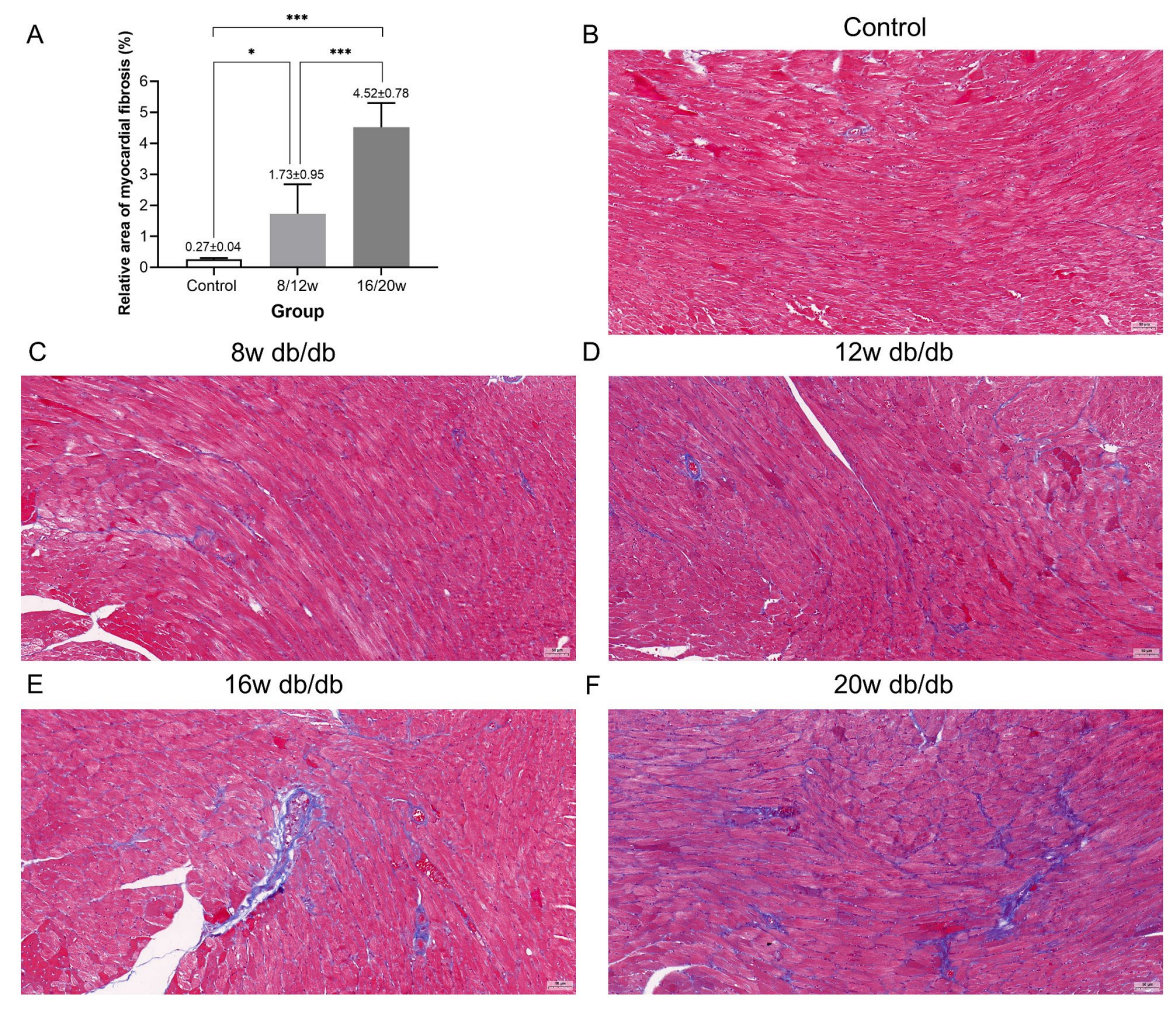
**Comparison of the degree of cardiac fibrosis (blue Masson staining) in db/db mice at different DCM stages (×200 times).** A: ^*^ indicates *P* < 0.05, ^***^ indicates *P* < 0.001; B: Control group db/+ mice; C: 8w db/db mice; D: 12w db/db mice; E: 16w db/db mice; F: 20w db/db mice

**Fig. 2:**
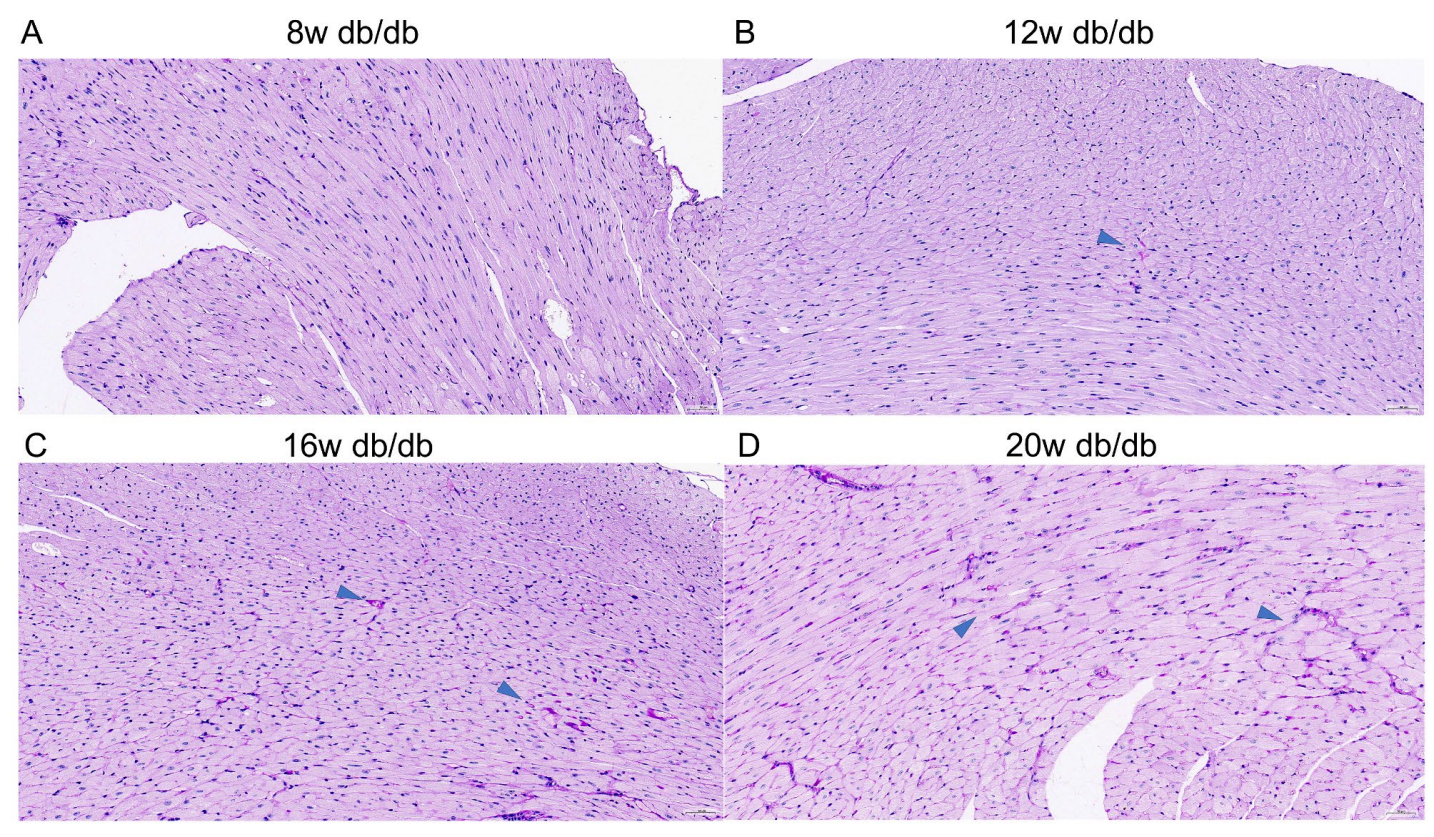
**The deposition of PAS-positive material in the heart of db/db mice at different DCM stages (×400 times).** A: 8w db/db mice (-); B: 12w db/db mice (+); C: 16w db/db mice (++); D: 20w db/db mice (+++); The blue arrows indicate deposition of PAS-positive material

**Fig. 3:**
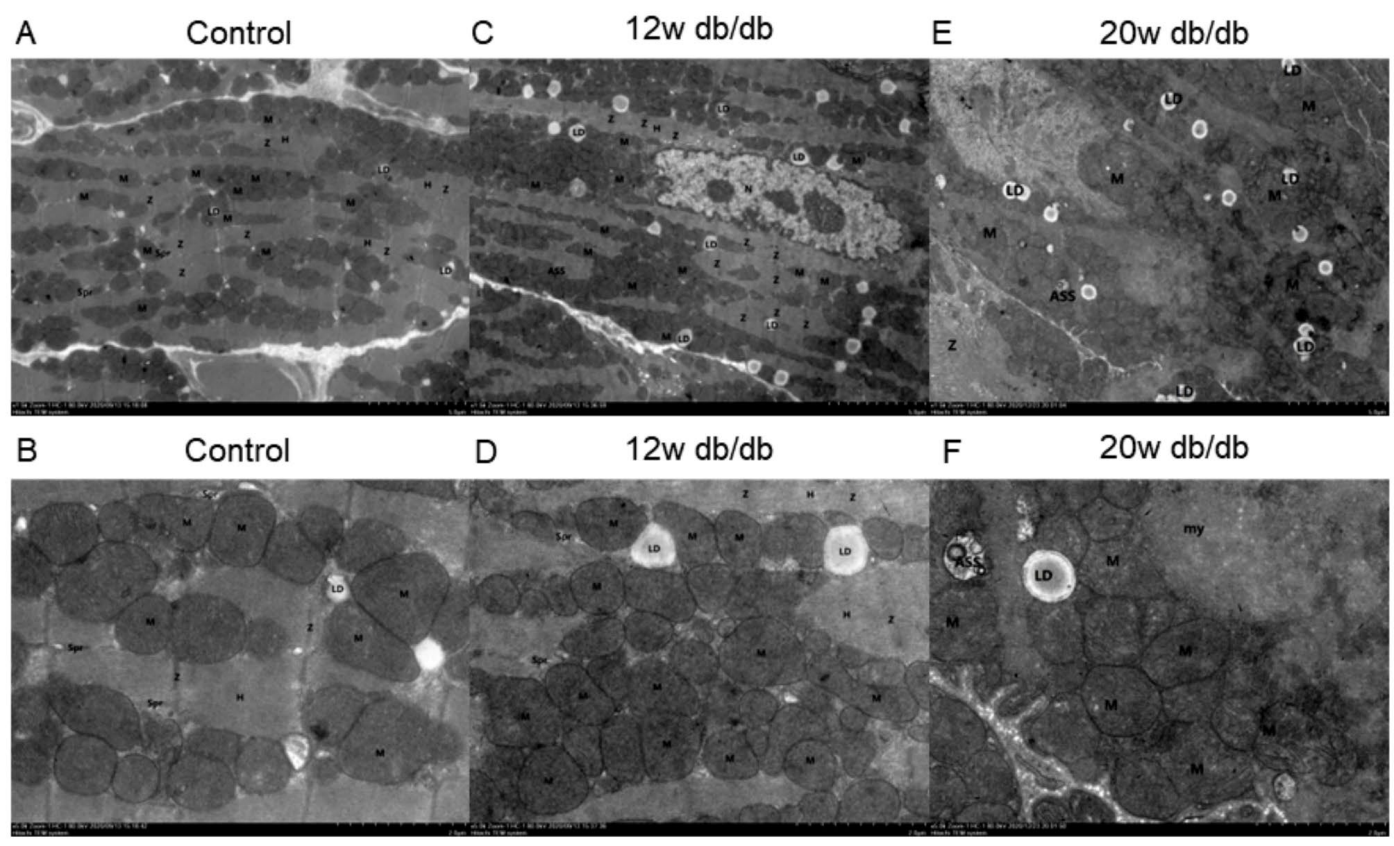
**Transmission electron microscopy images of myocardial ultrastructure in control db/+ mice (A-B), 12w (C-D) and 20w (E-F) db/db mice. Figures A, C, E (×1500), Figures B, D, F (×5000).** A-B: Myofibrils are regularly arranged and the sarcomere length is uniform. Mitochondria (M) structure is clear, with intact membrane and cristae; sarcoplasmic reticulum (Spr) has no obvious expansion; Z line (Z) is arranged continuously and regularly, without obvious asymmetric widening; complete and continuous H-band (H) structure can be seen; there are a few small and scattered lipid droplets (LD). C-D: Moderate hypertrophy of cardiomyocytes with mild steatosis, myofibril rupture and dissolution. Mitochondria (M) are slightly swollen, the electron density is slightly reduced, the intramembrane matrix is dissolved, and the cristae are broken and reduced; the sarcoplasmic reticulum (Spr) is slightly expanded; the Z line (Z) structure is blurred and discontinuous; the H band (H) structure disappears, and the structure of thick and thin myofilaments is loose; the number of lipid droplets (LD) is relatively high and widely distributed; there is a few autophagolysosomes (ASS) can be observed. E-F: Cardiomyocytes are obviously enlarged with steatosis, and myofibrils are disordered. Mitochondria (M) are moderately expanded, the outer membrane is blurred, the cristae disappear, and abnormal aggregation increases; the sarcoplasmic reticulum (Spr) is expanded; the structure of Z line (Z) and H band (H) is blurred and invisible, and some thick and thin filaments are broken; the number of lipid droplets (LD) is significantly increased; there are several autophagy lysosomes (ASS) can be seen in the field

The degree of myocardial fibrosis (blue Masson staining) in 16/20w db/db mice was significantly higher than that in 8/12w db/db mice and the control group (both *P* < 0.001, Fig. 1). The deposition of PAS-positive material (focal purple-red material) in the myocardial interstitium of 16/20w db/db mice was more obvious (++ ~ +++) than that in 8/12w db/db mice (- ~ ++) (Fig. 2), and the control group had no obvious PAS-positive material deposition (-). Under the transmission electron microscope, the myocardial cell hypertrophy and steatosis in 16/20w db/db mice were more obvious compared with the 8/12w db/db mice, and the abnormality of the intracellular organelles and ultrastructure, especially the mitochondrial structure, was also more prominent (Fig. 3).

### The level of myocardial glucose metabolism in different DCM stages and its relationship with left ventricular function

**Fig. 4:**
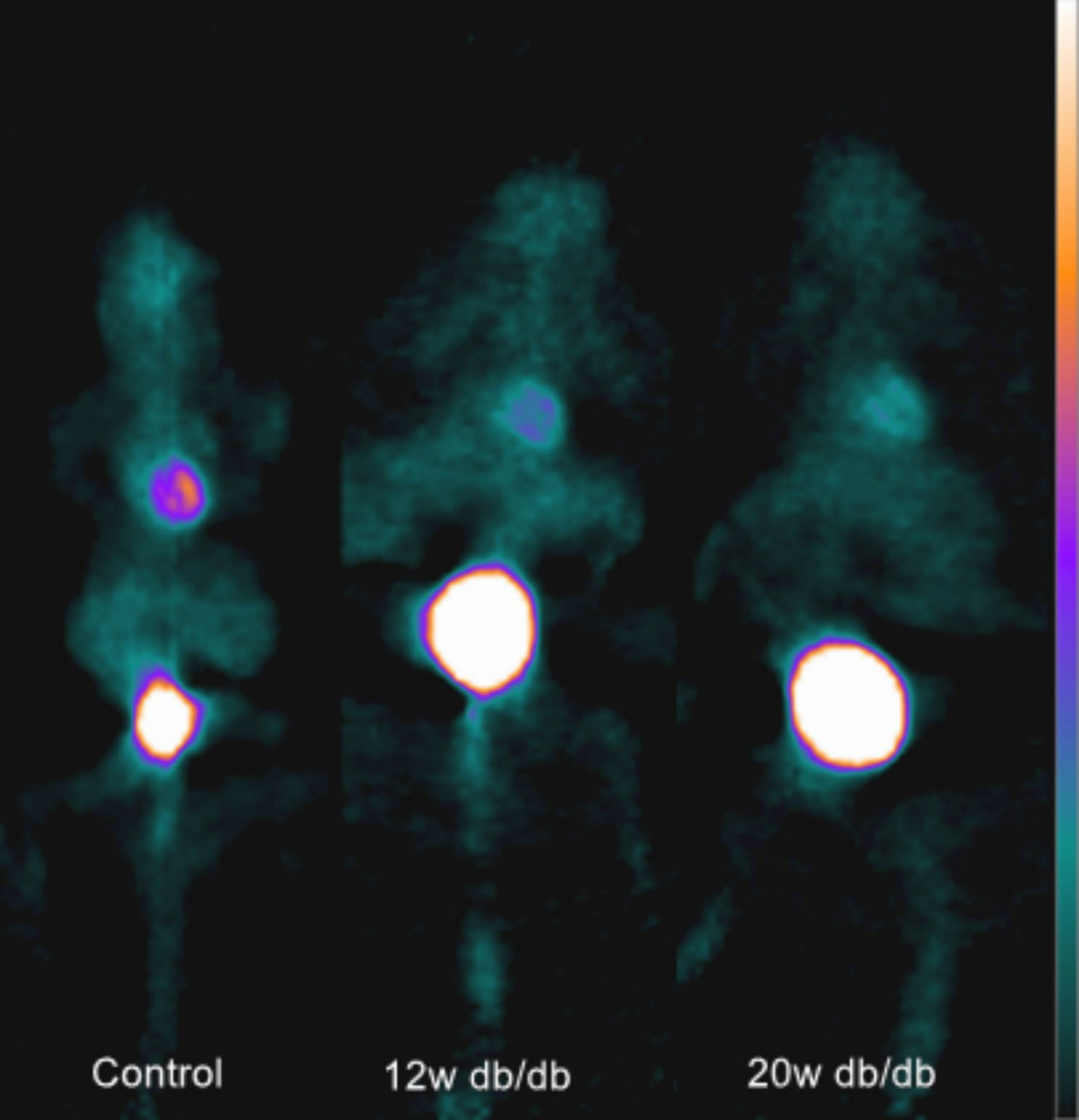
MicroPET images in control db/+ mice, 12w and 20w db/db mice

**Fig. 5:**
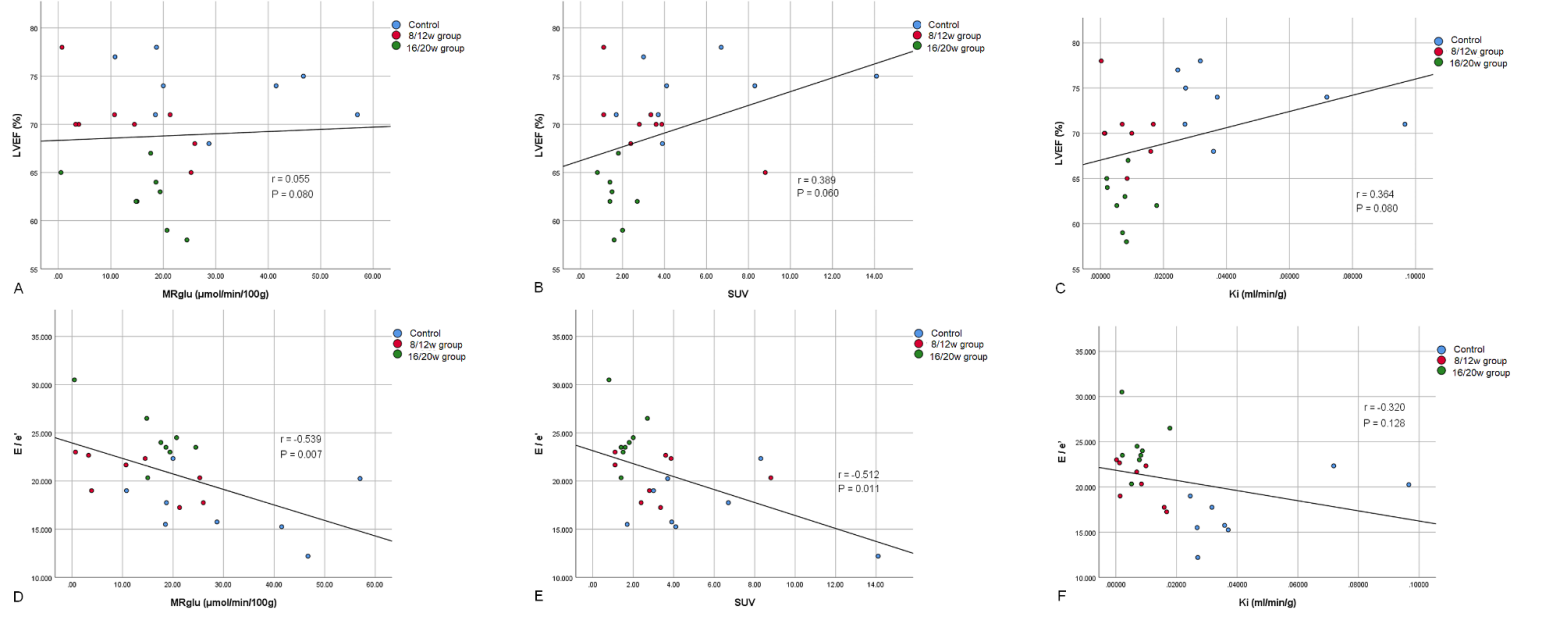
**The relationship between myocardial glucose metabolism and cardiac function.** (A) The correlation between MRglu and LVEF; (B) The correlation between SUV and LVEF; (C) The correlation between Ki and LVEF; (D) The correlation between MRglu and E/e’; (E) The correlation between SUV and E/e’; (F) The correlation between Ki and E/e’

As shown in Tables 2 and Fig. 4, the myocardial MRglu and Ki of 8/12w or 16/20w db/db mice were significantly reduced than those in the control group (*P* = 0.000 ~ 0.029), while the myocardial SUV was not significantly reduced than in controls until 16/20 weeks (*P* = 0.007). As shown in Fig. 5, MRglu and SUV were moderately negatively correlated with E/e’ ratio, with the correlation coefficients r of -0.539 and − 0.512, and *P* were 0.007 and 0.011, respectively. MRglu and SUV had no significant correlation with LVEF (*P* > 0.05). Ki showed no significant correlation with LVEF or E/e’ ratio (*P* > 0.05).

**Table 2:** Changes of left ventricular function and myocardial glucose metabolism in diabetic mice

	FBG (mmol/L)	MRglu (µmol/min/100 g)	Ki (ml/min/g)	SUV
Control (n = 8)	7.0 ± 2.0	30.2 ± 16.3	0.044 ± 0.026	5.7 ± 4.0
8/12w (n = 8)	19.9 ± 6.4	13.2 ± 10.2	0.008 ± 0.006	3.4 ± 2.4
^a^ *P*	< 0.001^*^	0.009^*^	< 0.001^*^	0.104
16/20w (n = 8)	25.8 ± 6.0	16.4 ± 7.1	0.007 ± 0.005	1.7 ± 0.6
^a^ *P*	< 0.001^*^	0.029^*^	< 0.001^*^	0.007^*^
^b^ *P*	0.035^#^	0.598	0.975	0.218

Note:

1. FBG: fasting blood glucose; MRglu: myocardial metabolic rate of glucose;Ki: glucose uptake rate constant; SUV: standardized uptake value

2. ^a^*P*: *P* value compared to Control; ^b^*P*: *P* value compared to 8/12w group; the differences are significant (*P* < 0.05) when compared with control group (^*^) and 8/12w group (^#^)

### Expression of myocardial glucose metabolism-related proteins in different DCM stages

**Fig. 6:**
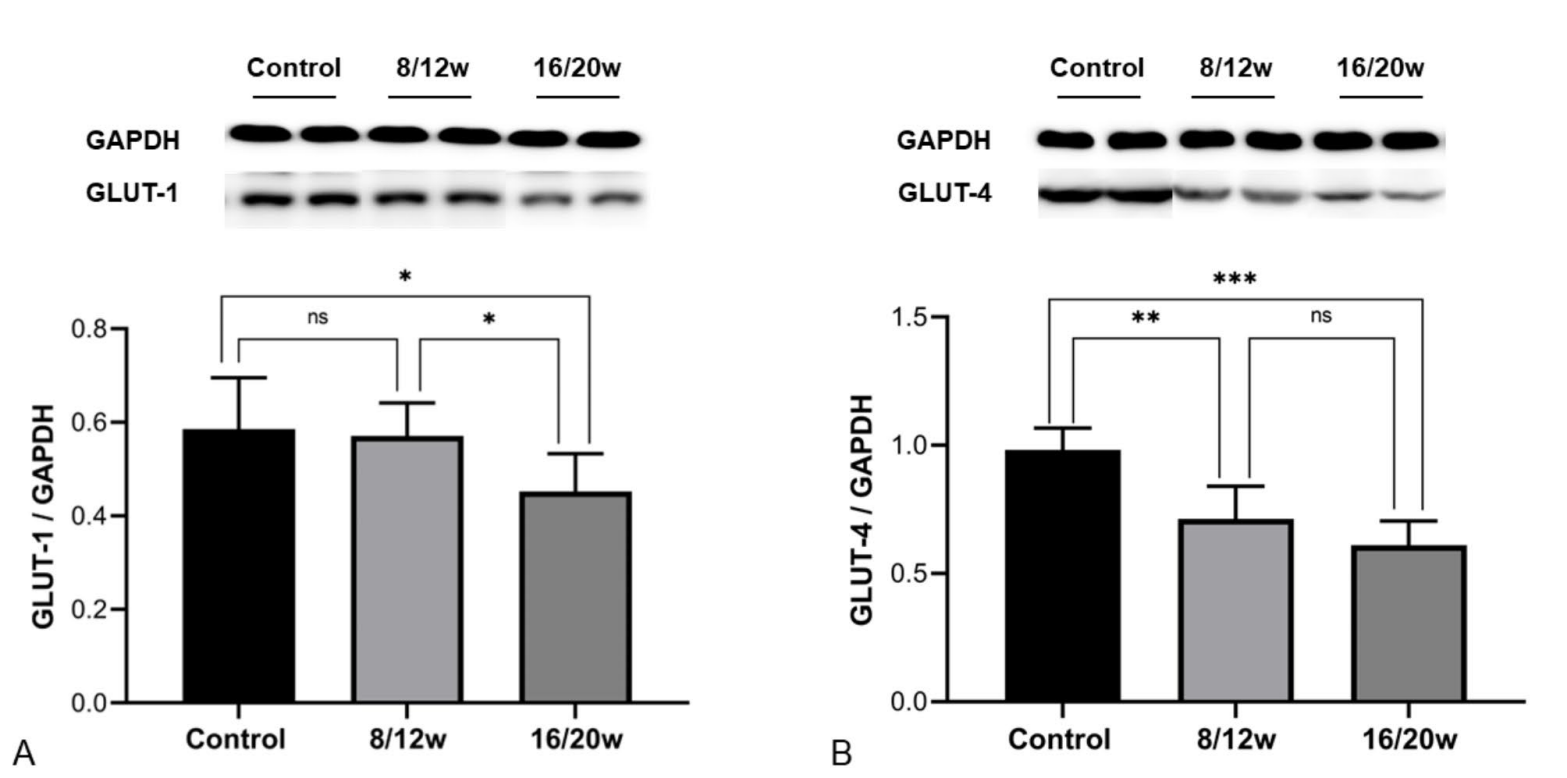
**The expression levels of GLUT-1 and GLUT-4 in the myocardium of diabetic mice were decreased.** A: GLUT-1 expression; B: GLUT-4 expression; ns, not significant; *, *P* < 0.05; **, *P* < 0.01; ***, *P* < 0.001

Compared with the control group, the expression of myocardial GLUT-4 of 8/12w db/db mice significantly decreased (0.71 ± 0.13 vs. 0.98 ± 0.09, *P* = 0.001), while the GLUT-1 did not change significantly (0.57 ± 0.07 vs. 0.59 ± 0.11, *P* = 0.788). The expression levels of both GLUT-1 and GLUT-4 in 16/20w db/db mice were significantly decreased than the controls (GLUT-1: 0.45 ± 0.08 vs. 0.59 ± 0.11, *P* = 0.027; GLUT-4: 0.61 ± 0.09 vs. 0.98 ± 0.09, *P* < 0.001), as shown in Fig. 6. Compared with 8/12w db/db mice, the GLUT-1 level in 16/20w db/db mice was significantly decreased (*P* = 0.045), while the GLUT-4 did not change significantly (*P* = 0.125), as shown in Fig. 6. Moreover, myocardial MRglu, Ki and SUV were positively correlated with myocardial GLUT-4 expression (MRglu: r = 0.537; Ki: r = 0.818; SUV: r = 0.491; *P* = 0.000 ~ 0.046), but not with myocardial GLUT-1 expression (*P* = 0.238 ~ 0.780).

**Fig. 7:**
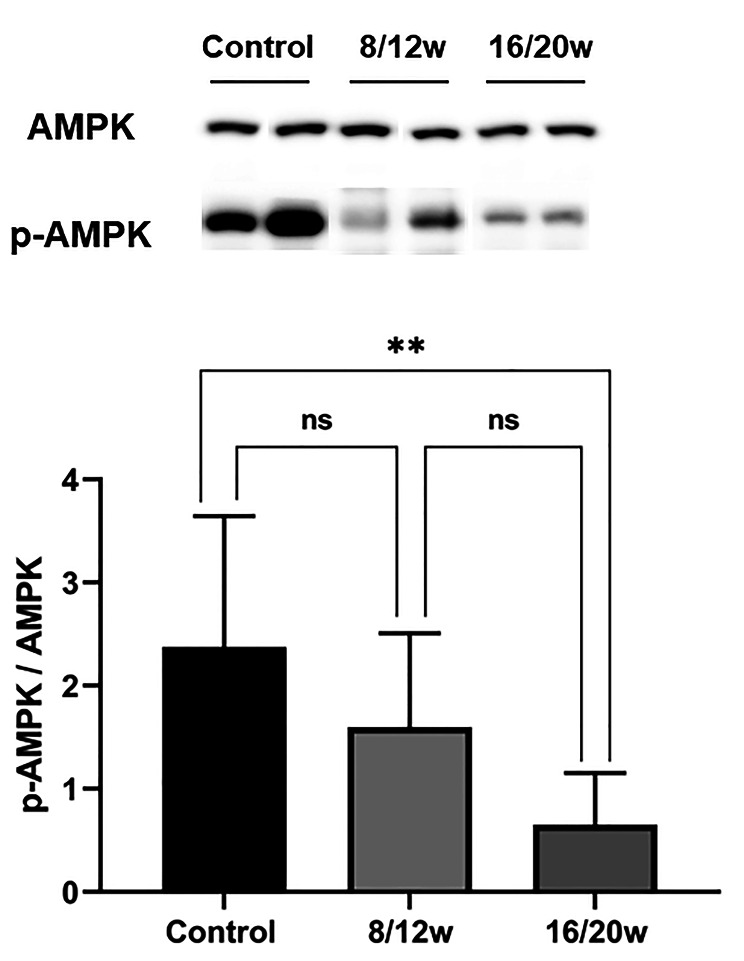
**The expression of p-AMPK in myocardium was decreased in diabetic mice.** ns, not significant; *, *P* < 0.05; **, *P* < 0.01; ***, *P* < 0.001

Compared with the control group, the level of myocardial p-AMPK protein in db/db mice (8 ~ 20w) was significantly reduced (1.13 ± 0.86 vs. 2.38 ± 1.29, *P* = 0.001). The myocardial p-AMPK protein level in 8/12w db/db mice was not significantly lower than the controls (1.60 ± 0.91 vs. 2.38 ± 1.29, *P* = 0.115), while the expression of myocardial p-AMPK protein in 16/20w db/db mice was significantly lower than that in the control group (0.65 ± 0.50 vs. 2.38 ± 1.29, *P* = 0.002) (Fig. 7).

## Discussion

Cardiac dysfunction is often clinically silent in diabetes and frequently is not detected until later stages. Left ventricular diastolic dysfunction is one of the earliest clinical manifestations of DCM and is detected earlier than clinically significant left ventricular systolic dysfunction [1, 21]. The E/e’ ratio is one of the indicators recommended by many guidelines for evaluating the left ventricular diastolic function, which has good reliability and repeatability [22, 23]. Like previous studies, only left ventricular diastolic dysfunction was observed in DCM stage 1. When the left ventricular systolic dysfunction was significant (DCM stage 2/3), the myocardium had been significantly remodeled and interstitial fibrosis was evident.

Cardiomyocyte hypertrophy, interstitial fibrosis and deposition of PAS-positive materials are typical pathological features of DCM. This study verified that the degree of myocardial fibrosis, PAS-positive material deposition, and myocardial ultrastructural damage, especially the abnormal mitochondrial structure, were more evident in DCM stage 2/3 (16/20w group) than those in DCM stage 1 (8/12w group). The mechanisms may be related to that chronic hyperglycemia can induce glycosylation of fibrinogen and albumin (the main components of PAS-positive substances [24]), the production of advanced glycation end products (AGEs), cross-linking of collagen molecules [25], loss of elasticity, myocardial stiffness, and decreased compliance [26]. Mitochondria are important places for integrating redox and metabolic signaling pathways [27], and their structural integrity and normal function are essential for maintaining normal myocardial function. Studies have shown that changes in myocardial substrate utilization, insulin resistance, and impaired insulin signaling, can disrupt mitochondrial morphology and function [4]. In this study, we found that compared with the control group, the myocardial mitochondrial outer membrane in diabetic mice was blurred, some cristae were broken and disappeared, and abnormal aggregation increased. Although the mitochondrial number was increased in diabetic mice, the mitochondria were small and often fragmented [28], which might be related to the decreased expression of mitochondrial fusion protein, and the imbalance between mitochondrial fusion and fission [28]. Mitochondrial fragmentation is a characteristic change in the ultrastructure of dysfunctional mitochondria.

Fatty acids, glucose, and ketone bodies are all substrates of myocardial energy metabolism, and the choice of substrates mainly depends on the availability of substrates, oxygen concentration, and load [10]. In 2004, Marc van Bilsen [29] proposed the concept of myocardial metabolic remodeling, which states that the changes in metabolic pathways of the heart can lead to abnormal cardiac structure and function. However, there are few studies on the dynamic evolution of myocardial metabolic remodeling after diabetes, and sensitive indicators to assess myocardial glucose metabolism levels in DCM are lacking. SUV is a semiquantitative index that can be used clinically to determine the level of myocardial glucose metabolism. However, our study found that myocardial SUV changes in the early stage of DCM were insignificant compared with the controls. MRglu and Ki were more sensitive than SUV in this study, while the exact relationship between myocardial MRglu and Ki and cardiac function remains controversial. Shoghi et al. [16] found that the myocardial glucose uptake rate of 14-week-old and 19-week-old Zucker diabetic rats were significantly lower than that of the control group, while the myocardial glucose uptake rate of 19-week-old diabetic rats was slightly higher than that of 14-week-old rats (*P* > 0.05). Our study found that the myocardial MRglu and Ki of the db/db mice were significantly lower than that in the control group. Although the myocardial MRglu of 16/20w group was slightly higher than that of 8/12w group, the difference was not significant, which was consistent with the study of Shoghi et al. [16].

Through microPET imaging, Brom et al. [13] found that the myocardial glucose uptake rate in diabetic rats was strongly positively correlated with the short-axis shortening rate of the left ventricle (reduction indicates abnormal left ventricular systolic function) (r = 0.91), and was significantly negatively correlated with the E-peak deceleration time (prolongation indicates decreased diastolic function) (r = -0.70). Our study found that MRglu and SUV were moderately negatively correlated with the E/e’ ratio (r = -0.539 and − 0.512, *P* = 0.007 and 0.011), and Ki showed no significant correlation with the E/e’ ratio. These results suggested that decreased myocardial glucose uptake is not the dominant factor of cardiac dysfunction, and reduced myocardial glucose metabolism may be the main factor. Therefore, targeted improvement of myocardial glucose metabolism can help improve cardiac function in diabetic patients.

GLUT-1 and GLUT-4 are the most abundant glucose transporters in the myocardium. GLUT-1 is mainly located on the plasma membrane and maintains the myocardial glucose uptake in a steady state. GLUT-4 mainly exists in intracellular vesicles and can be translocated to the plasma membrane when cells are stimulated by insulin. GLUT-4-mediated glucose transport is an essential mechanism of myocardial glucose uptake that can be tightly regulated by environmental changes [30]. Unlike the animal models of heart failure (GLUT-1 expression increases and GLUT-4 expression decreases), Yang et al. [31] found that the expressions of GLUT-1 and GLUT-4 in diabetic mice were significantly lower than those in the control group, which is consistent with our findings. However, our study also found that the timings of downregulation of GLUT-4 and GLUT-1 during diabetes differed. The mechanism may be related to insulin resistance in the myocardium, and the expression of insulin-dependent GLUT-4 is more susceptible to environmental factors [32]. More importantly, this study also found that myocardial MRglu, Ki and SUV measured by microPET positively correlated with myocardial GLUT-4 expression. This result provides a theoretical basis for using radionuclide molecular imaging to non-invasively monitor myocardial glucose metabolism and metabolic remodeling in diabetic patients.

**Fig. 8:**
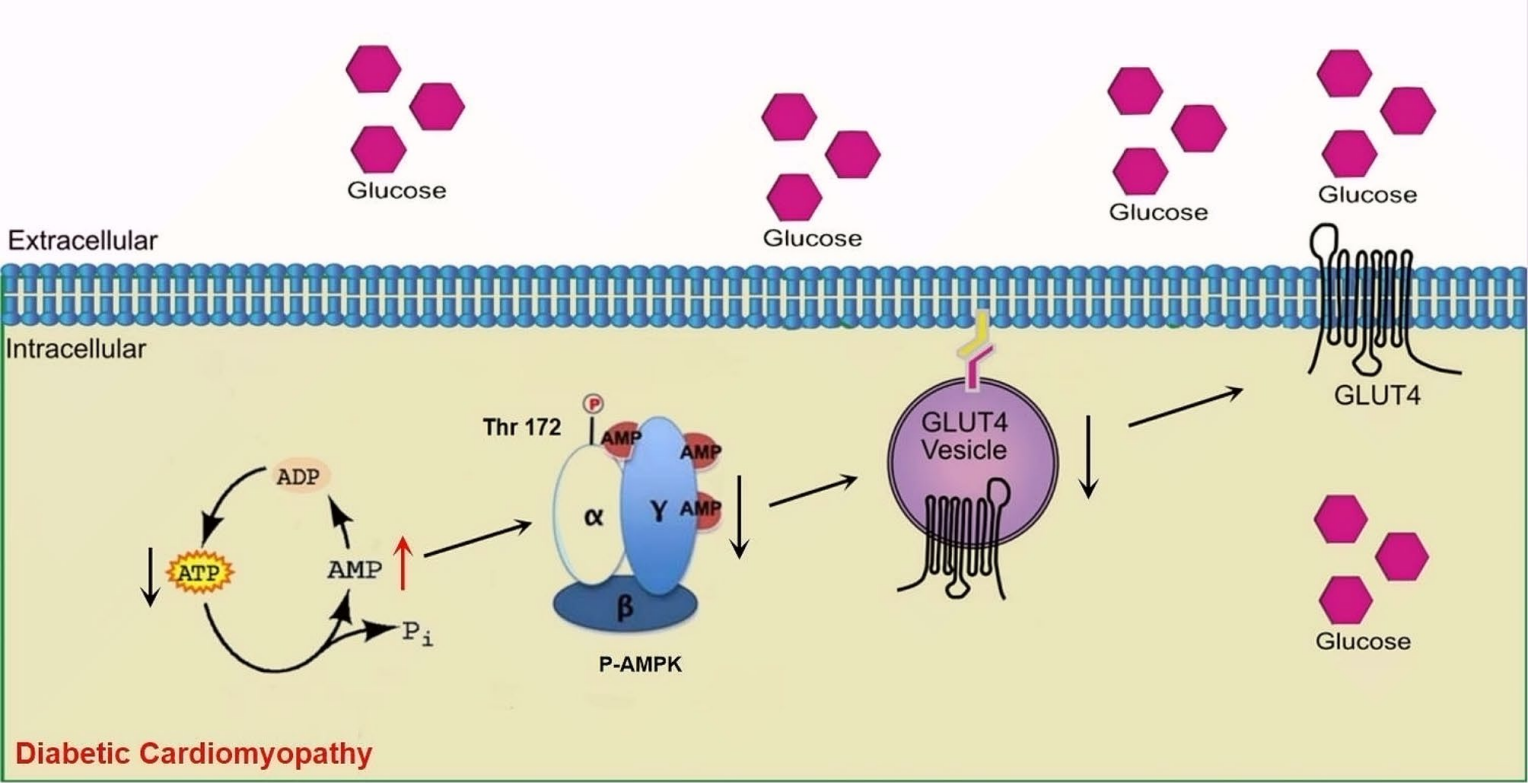
** A schematic diagram of the molecular mechanisms involved in this study leading to decreased myocardial glucose metabolism.** During the development of DCM, myocardial AMPK phosphorylation is inhibited, and its activity is reduced, accompanied by reduced GLUT-4 membrane translocation and myocardial glucose metabolism

AMPK is a critical factor in the regulation of cellular energy metabolism. It consists of three subunits, α, β, and γ. In myocardial tissue, the γ subunit of AMPK combines with AMP and ATP to form a complex and undergoes a conformational change, which promotes the phosphorylation of an essential residue (threonine 172) of α subunit, leading to AMPK activation [33]. Studies have shown that, during the development of DCM, myocardial AMPK phosphorylation is inhibited, and its activity is reduced, accompanied by reduced GLUT-4 membrane translocation [31], Fig. 8. This result also confirmed that in addition to the insulin-mediated GLUT-4 regulatory pathway (phosphatidylinositol 3-kinase/protein kinase B signaling pathway), the insulin-independent signaling pathways (SIRT3/AMPK pathway, etc.) also play a role in the development of DCM [34].

### Limitations

This study also has the following limitations. First, the hyperinsulinemic normoglycemic clamp method is the “gold standard” for evaluating tissue insulin sensitivity. This method can maintain blood glucose fluctuations less than 5% by administering exogenous insulin and glucose intravenously. Therefore, the impact of fasting blood glucose differences between experimental animals on the experimental results is minimized. However, this method is challenging for small animals and requires special equipment. For this reason, we did not use the hyperinsulinemic normoglycemic clamp method before microPET imaging. Second, this study’s calculation of MRglu and Ki adopted the Patlak graphical analysis. Although this method avoids repeated tail vein blood sampling to obtain the input function (generally, the physiological blood volume of mice is only 5.85 ml/100 g body weight), the resolution of the instrument, partial volume effect, and counting overflow may all have a particular impact on the image delineation. Third, isoflurane anesthesia may affect myocardial glucose metabolism [35]. However, since isoflurane anesthesia was used in all experimental mice for microPET imaging, it would not affect detecting differences in myocardial glucose metabolism between groups.

## Conclusion

In this study, the dynamic evolution of DCM was observed by echocardiography, histopathology and transmission electron microscopy. DCM functional phenotypes were divided. Meanwhile, ^18^ F-FDG microPET dynamic imaging was used to identify the abnormal myocardial glucose metabolism corresponding to different DCM functional phenotypes. It has been found that abnormal myocardial glucose metabolism can occur early with the DCM phenotypes changes. MRglu is a sensitive index that reflects the changes of myocardial glucose metabolism in DCM and correlates well with left ventricular diastolic function and GLUT-4 expression.

## Electronic supplementary material

Below is the link to the electronic supplementary material.

Supplementary Material 1

## Data Availability

The datasets used and analyzed during the current study are available from the corresponding author on reasonable request.

## Abbreviations

DCM: Diabetic cardiomyopathy
Ki: Glucose uptake rate constant
LV: Left ventricular
FDG: ^18^ F-deoxyglucose
LVEDD: Left ventricular end-diastolic diameter
LVESD: Left ventricular end-systolic diameter
LVEF: Left ventricular ejection fraction
MRglu: Myocardial metabolic rate of glucose
SUV: Standardized uptake value
VOI: Volume of interest
