# Supplementary material for: Dynamic evolution and mechanism of myocardial glucose metabolism in different functional phenotypes of diabetic cardiomyopathy — a study based on 18 F-FDG microPET myocardial metabolic imaging
Source: Diabetol Metab Syndr. 2023 Apr 1;15:64. doi: 10.1186/s13098-023-01038-5 (PMC10067248; doi:10.1186/s13098-023-01038-5)
Supplement: Supplementary file 1 — Supplementary Material 1 [file 13098_2023_1038_MOESM1_ESM.pdf]

Dynamic evolution and mechanism of myocardial glucose metabolism in different functional phenotypes of diabetic cardiomyopathy — A study based on ^{18}F-FDG microPET myocardial metabolic imaging

Xiaoliang Shao[1,2], Yaqi Liu[1,2], Mingge Zhou[1,2], Min Xu[3], Yuqi Chen[1,2], Hongbo Huang[4], Jianguo Lin[4], Yuetao Wang[1,2*]

[1] Department of Nuclear Medicine, the Third Affiliated Hospital of Soochow University, Changzhou 213003, China

[2] Clinical Translational Institute for Nuclear Medicine and Molecular Imaging of Soochow University, Changzhou 213003, China

[3] Echocardiography Division in Department of Cardiology, the Third Affiliated Hospital of Soochow University, Changzhou 213003, China

[4] NHC Key Laboratory of Nuclear Medicine, Jiangsu Key Laboratory of Molecular Nuclear Medicine, Jiangsu Institute of Nuclear Medicine, Wuxi 214063, China

***Correspondence**

Yuetao Wang, MD

Department of Nuclear Medicine, the Third Affiliated Hospital of Soochow University, Changzhou 213003, China

Clinical Translational Institute for Nuclear Medicine and Molecular Imaging of Soochow University, Changzhou 213003, China

Email: yuetao-w@163.com; Fax: 86-519-86621235; ORCID: 0000-0003-2859-8625

Fig 6. Supplementary file

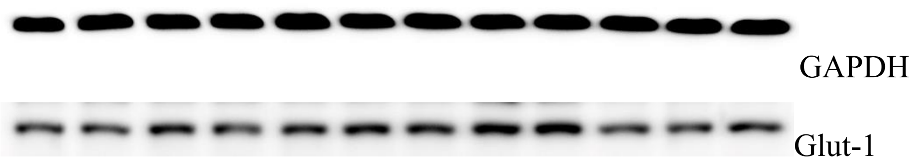

From left to right：db/+-8w-1, db/+-8w-2, db/db-8w-1, db/db-8w-2, db/db-8w-3, db/db-8w-4, db/db-12w-1, db/+-12w-1, db/+-12w-2, db/db-12w-2, db/db-12w-3, db/db-12w-4.

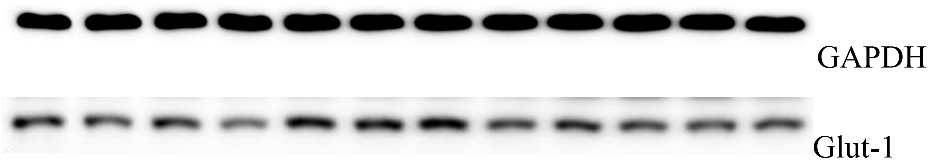

From left to right： db/db-16w-1, db/db-16w-2, db/db-16w-3, db/db-16w-4, db/+-16w-1, db/+-16w-2, db/+-20w-1, db/+-20w-2, db/db-20w-1, db/db-20w-2, db/db-20w-3, db/db-20w-4.

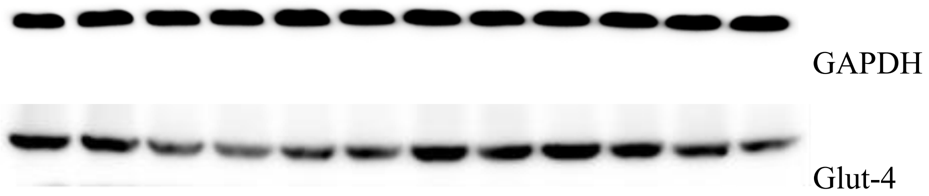

From left to right：db/+-12w-1, db/+-12w-2, db/db-12w-1, db/db-12w-2, db/db-12w-3, db/db-12w-4, db/+-8w-1, db/+-8w-2, db/db-8w-1, db/db-8w-2, db/db-8w-3, db/db-8w-4.

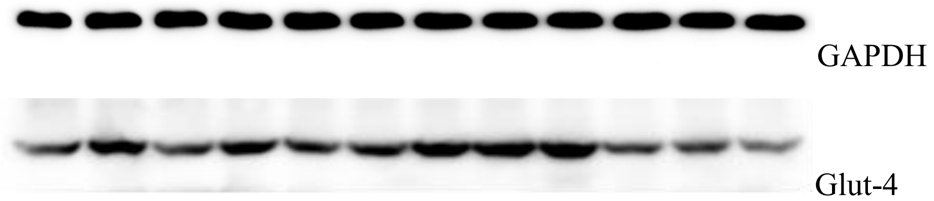

From left to right：db/+-16w-1, db/+-16w-2, db/db-16w-1, db/db-16w-2, db/db-16w-3, db/db-16w-4, db/+-20w-1, db/+-20w-2, db/db-20w-1, db/db-20w-2, db/db-20w-3,

db/db-20w-4.

Fig 7. Supplementary file

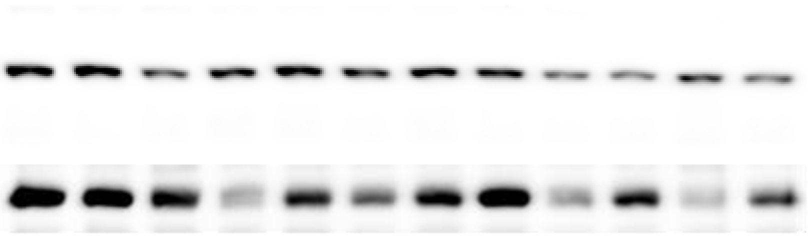

AMPK

pAMPK

From left to right：db/+-8w-1, db/+-8w-2, db/db-8w-1, db/db-8w-2, db/db-8w-3, db/db-8w-4, db/+-12w-1, db/+-12w-2, db/db-12w-1, db/db-12w-2, db/db-12w-3, db/db-12w-4.

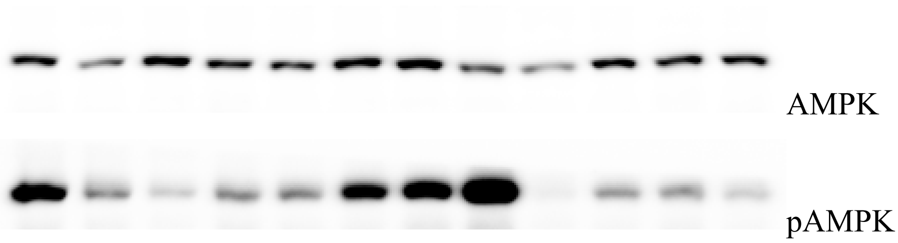

AMPK

pAMPK

From left to right：db/+-16w-1, db/+-16w-2, db/db-16w-1, db/db-16w-2, db/db-16w-3, db/db-16w-4, db/+-20w-1, db/+-20w-2, db/db-20w-1, db/db-20w-2, db/db-20w-3, db/db-20w-4.
